# Physicians’ Compliance with COVID-19 Regulations: The Role of Emotions and Trust

**DOI:** 10.3390/healthcare10030582

**Published:** 2022-03-20

**Authors:** Shosh Shahrabani, Shiran Bord, Hanna Admi, Michael Halberthal

**Affiliations:** 1 The Economics and Management Department, The Max Stern Yezreel Valley College, Emek Yezreel 1930600, Israel; 2 Health Systems Management Department, The Max Stern Yezreel Valley College, Emek Yezreel 1930600, Israel; shiranb@yvc.ac.il; 3 Head of Master’s Program in Nursing Leadership, The Max Stern Yezreel Valley College, Emek Yezreel 1930600, Israel; hannaa@yvc.ac.il; 4 Director General & CEO, Rambam Health Care Campus, Haifa 3109601, Israel; m_halberthal@rambam.health.gov.il

**Keywords:** COVID-19, preventive health, physicians, perceptions, emotions, trust

## Abstract

(1) Background: Medical teams are at the forefront of the COVID-19 pandemic. Decision making among medical staff is important for promoting and maintaining the health of patients and staff. This study examines factors associated with physicians’ decision making and preventive behavior during the COVID-19 pandemic in Israel. (2) Methods: An online survey was conducted among 187 Israeli physicians in April and May 2020 during the COVID-19 pandemic. The questionnaire included the levels of physicians’ perceived threat and perceived risk during the epidemic, trust in the health system, emotions, and the degree of compliance with hygiene rules and mandated behaviors. (3) Results: Most physicians reported complying with the rules of hygiene at work (73%) and full compliance with Ministry of Health guidelines (61%). The findings show that higher levels of trust, positive emotions, and threat and risk perceptions were associated with a higher degree of compliance with Ministry of Health guidelines and more careful decision making among physicians. (4) Conclusions: Levels of trust in the health system and positive emotions among healthcare staff during the pandemic are related to careful adherence to guidelines. Taking steps to maintain physical and mental health among healthcare staff is important for their functioning and for maintaining public health.

## 1. Introduction

Across the globe, physicians and other healthcare workers (HCWs) are on the frontline of the battle against the COVID-19 pandemic, while they are at high risks of contracting COVID-19 [1]. In fact, HCWs work long and intensive hours during the pandemic and many have developed occupational burnout as a result of this excessive burden and continuous stress [2]. A key element in battling the pandemic is compliance with the guiding principles established by the World Health Organization (WHO) and the health ministry of each country.

Primary preventive measures include wearing masks that cover the mouth and nose, regular hand washing, using hand sanitizers, and physical distancing [3,4]. During the first wave of the COVID-19 epidemic in Israel, the Israeli Ministry of Health (MOH) issued guidelines for HCWs. As part of these guidelines, HCWs were asked to wear masks while treating patients in the emergency room as well as disposable gowns and gloves. In addition, HCWs were instructed to ask patients “suspected” of having COVID-19 to wear masks while in emergency rooms and in the hospital [5].

During this period, MOH also issued additional general guidelines for all citizens (guidelines for behavior at home). These guidelines were aimed at minimizing contact and maintaining physical distancing and optimal hygiene, including frequent hand washing [6]. An Israeli study conducted during the first wave of the COVID-19 epidemic in Israel among adults over the age of 60 found that public compliance with the MOH guidelines was high. More than 70% of the respondents reported strictly adhering to the regulations [7]. Compliance with the preventive measures according to the guidelines is particularly crucial for HCWs, especially at work, in order to protect themselves, their patients, and their families from infection.

Several studies conducted during the COVID-19 pandemic indicate that preventive measures taken by HCWs against COVID-19 were assessed at a relatively desirable level [8]. Yet other studies point to insufficient compliance with guidelines [9,10]. For example, in India, resident doctors and other paramedical staff exhibited lower adherence to preventive practices than nurses and senior doctors [10]. Moreover, previous studies indicated that some HCWs were hesitant to take the COVID-19 vaccine [11,12]. The worldwide prevalence of COVID-19 vaccination hesitancy among healthcare workers ranged from 4.3% to 72% [11]. Yet, while previous studies mainly examined HCWs’ acceptance of COVID 19 control measures, HCWs encompass various types of professional workers who may differ in their attitudes and decision making. The current study adds to the existing literature by focusing on physicians’ compliance with regulation during the pandemic.

Previous studies have shown that threat and risk perceptions are associated with preventive behavior. According to the Health Belief Model (HBM) [13], preventive health behavior is associated with the perceived severity of the disease and with people’s perceived susceptibility to the disease. Perceived disease severity and susceptibility define an individual’s threat perceptions regarding a particular disease. Perceived severity refers to beliefs regarding the negative effects of contracting the infection, while perceived susceptibility refers to beliefs regarding vulnerability to the infection [13].

Recent empirical studies conducted during the COVID-19 pandemic have confirmed the predictions of the health belief model [14,15,16]. For example, perceived severity and perceived susceptibility were important predictors of coronavirus prevention behaviors in China [16,17]. Yet most of these studies were conducted among the general population, while only a few studies examined the relationship between HBM and preventive behavior and, particularly, vaccine intention during the pandemic among healthcare workers [17,18]. The results of these studies with respect to the impact of perceived threat on preventive measures were not consistent. For example, Yu et al. [18] found that, among healthcare workers in China, perceived severity exhibited a significant association with the intention to be vaccinated against COVID-19, while in the case of perceived susceptibility, this association was not significant. A study by Maraqa et al. [19] among Palestinian HCWs showed that perceived COVID-19 severity and susceptibility were among the reasons cited for getting vaccinated. The majority of HCWs considered themselves vulnerable to COVID-19 and wanted to avoid transmitting COVID-19 to their families or patients.

The results of previous studies conducted in several countries were not consistent. Therefore, the current study adds to the existing literature by focusing on the effect of the HBM construct of threat perception on precautionary actions taken by physicians in the healthcare system in Israel during the pandemic. Examining perceptions and preventive behavior among HCWs in different countries during the pandemic can shed light on decision making among HCWs.

Trust in the authorities and, particularly, in the ministry of health in each country is another factor that may be related to making decisions about preventive behavior. Trust in government represents “the confidence or satisfaction of people with government performance and the perceived credibility of government” [20,21]. Recent studies conducted during the COVID-19 pandemic confirm that public trust in the government is important for encouraging the implementation of social policies that rely on behavioral adherence among the public [20,22]. Recent studies also mentioned two groups distinguished by their individual characteristics relative to their compliance with COVID-19 public health guidelines [23,24]: Group A, the Eudaimonic group, comprises individuals who exhibit high levels of adherence to public health guidelines, have higher levels of trust in the government, are more threatened by the pandemic, and are more likely to be older and female. Group B, the Hedonic group, includes people who demonstrate low levels of adherence to public health guidelines, show political conservatism, believe in conspiracy theories, and are less threatened by the pandemic, yet most of these studies focused on public trust and other attributes of public attitudes and behavior. The current study focuses on HCWs. It examines how physicians’ trust in the conduct of the Ministry of Health (MOH) and the instructions it issued to the public during the pandemic are related to physicians’ own compliance with preventive health guidelines.

The pandemic elicited diverse emotions, both negative and positive, among HCWs. Several theories of preventive health behavior suggest that emotions play a central role. For example, the cognitive-social health information processing (C-SHIP) model [25] contends that affective states are related to preventive health behaviors. In line with these theories, Chapman and Coups [26] found that anticipated and experienced emotions, such as worry and regret, had implications for subsequent vaccination decisions. Therefore, one hypothesis of the current study is that the intensity of physicians’ emotions during the pandemic is related to their decision making regarding preventive health behavior.

Moreover, previous studies indicate that, in the wake of major events, women generally report higher levels of fear and self-risk assessment than men [27,28,29,30]. For example, Finucane et al. [31] found that women’s risk estimates of a wide range of hazards were higher than those of men (e.g., hazardous activities, technologies such as blood transfusions, violent crimes, and vaccines). In the context of the COVID-19 pandemic, Dryhurst et al. [32] found a gender effect, such that men had lower COVID-19 risk perceptions than women. In addition, previous studies found that, during the COVID-19 pandemic, men had lower intentions to adopt preventive behaviors than women [33,34]. For example, Capraro and Barcelo [34] found that men had lower intentions than women to cover their faces, although this difference practically disappeared in countries where face masks were mandatory. Nevertheless, none of these previous studies examined gender differences in the context of emotions, perceived risk, perceived threat, and preventive behavior among HCWs and physicians. Our study fills this void.

The current study adds to the existing literature in three ways: (a) It examines the level of preventive measures among female and male physicians who were on duty during the first wave of the COVID-19 pandemic in Israel. The existing literature does not include enough studies conducted among physicians during the pandemic. (b) It examines the factors that affected physicians’ decisions about whether to comply with the preventive action guidelines issued by MOH during the pandemic: trust in the MOH, emotions aroused during the pandemic and physicians’ health beliefs. (c) It examines whether the factors affecting preventive health behavior differ between men and women. Understanding what motivates HCWs to take precautionary actions is important in planning further steps to increase their compliance and reducing infection rates.

### Hypotheses

Based on the literature review, we hypothesize the following.

**Hypothesis** **1** **(H1).** *Physicians who have higher levels of threat and risk perceptions in the context of the pandemic will exhibit higher levels of preventive behavior.*

**Hypothesis** **1a** **(H1a).** *That Hypothesis 1 is null. No relationships will be found between threat and risk perceptions and preventive behavior.*

**Hypotheses** **2** **(H2).** *Physicians who have higher levels of trust in the MOH and the healthcare system will exhibit higher levels of preventive behavior.*

**Hypothesis** **2a** **(H2a).** *That Hypothesis 2 is null. No relationships will be found between trust in the MOH and the healthcare system and preventive behavior.*

**Hypotheses** **3** **(H3).** *Physicians who have higher levels of emotions (either negative or positive) will exhibit higher levels of preventive behavior.*

**Hypothesis** **3a** **(H3a).** *That Hypothesis 3 is null. No relationships will be found between levels of emotions (either negative or positive) and preventive behavior.*

**Hypotheses** **4** **(H4).** *Female physicians will have higher levels of negative emotions, higher threat and risk perceptions, and higher levels of preventive behaviors than male physicians.*

**Hypothesis** **4a** **(H4a).** *That Hypothesis 4 is null. No gender differences will be found in levels of negative emotions, threat and risk perceptions, and preventive behavior.*

## 2. Materials and Methods

The current study is a cross-sectional online survey of Israeli physicians conducted by a professional polling company using a convenience sampling method. The polling company recruited respondents by approaching professional physician associations and asking them to distribute the survey link to the association’s members. To reduce possible selectivity bias, several reminders were sent to the members of the professional physician associations. In addition, the polling company recruited respondents by using snowball sampling, such that one physician referred the polling company to others.

The Institutional Review Board of the Yezreel Valley College approved the current study. The study was conducted between 21 April and 15 May 2020, toward the end of the first wave of the COVID-19 pandemic in Israel. (During the period of the survey, an average of 200 people were infected each week during April, while by the end of May 2020 50 new infections were reported each week (https://github.com/CSSEGISandData/COVID-19, accessed on 20 October 2021).

### 2.1. Participants

Table 1 shows the demographic and other characteristics of the sample by gender.

Participants in this study included 200 physicians between the ages of 27 and 77 (M = 41.27 years, SD = 10.60). About half the participating physicians were men (49%) and most were Jewish (86%). The majority were born in Israel (75%); married or in an intimate relationship (89%); secular (71%); and reported above average economic status (82%). None of the demographic characteristics reflected major gender differences between male and female physicians. Participants’ seniority in medical practice ranged between 0.5 and 50 years (M = 12.54 years; SD = 11.59). Table 1 also shows that most of the participating physicians were employed in hospitals (91%), and about one-fifth held managerial positions (20%).

### 2.2. Questionnaire

In the current study, we used a questionnaire that included several parts. These parts were based on questionnaires that were validated in previous studies (see below). The questionnaire was translated into Hebrew by one of the authors and then back-translated by an English editor. In the first stage, face validity and intelligibility were examined by each co-author. In the second stage, the questionnaire was sent to three experts to ensure expert validity. In the third stage, a pilot questionnaire was administered to ten physicians. The final format was developed after improvements were made. Moreover, we examined the internal reliability of each part of the questionnaire by using Cronbach’s alpha (see Table 2). All alpha values were above 0.7.

The Qualtrics platform was used to construct the questionnaire, which is provided in Appendix A. The questionnaire included the following parts:

1. Socio-demographic information and personal details, including position at work (managerial/non-managerial), place of work (hospital/community service), and seniority as a physician (years).
2. Physicians’ compliance with MOH regulations (henceforth, compliance). The compliance variable was measured on a 7-point scale (1 = not at all; 7 = always). Participants were asked to indicate the frequency with which they performed three preventive actions during the coronavirus crisis (e.g., “More meticulous than usual in complying with MOH-issued COVID-19 regulations”). The compliance variable was computed as the participants’ mean response to these three items.
3. Perceived threat was measured based on the seven-item subscale of the validated Hebrew version of HBM [35]. Participants were asked to indicate the levels of their agreement with the seven items on a 7-point scale (1 = “strongly disagree” to 7 = “strongly agree”) (e.g., “The thought of getting COVID-19 scares me”). The perceived threat variable was computed as the participants’ mean response to the seven items.
4. Perceived risk during the COVID-19 pandemic was measured based on the Domain-Specific Risk-Taking (DOSPERT) scale [36]. Participants were asked to indicate the level of danger they feel regarding nine items. Each item was rated on a 7-point scale ranging from 1 = “not dangerous at all” to 7 = “extremely dangerous” (e.g., “treating a COVID-19 patient without protective equipment”). The perceived risk variable was computed as the participants’ mean response to the nine items.
5. Positive and negative emotion levels were measured using the Positive and Negative Affect Schedule (PANAS) questionnaire [37]. Participants were asked to indicate the levels of negative and positive emotions they felt during the last week on a 7-point scale ranging from “not at all” (1) to “extremely” (7). The emotions variable was computed as the participants’ mean response to the positive and negative items, separately.
6. Trust in the healthcare system was based on the research of Dugan et al. and Egede and Ellis [38,39]. Participants were asked to indicate their level of agreement with nine items on a 7-point scale ranging from 1 = do not agree at all to 7 = fully agree (e.g., “I have confidence in the medical information published by the Ministry of Health”). The trust variable was computed as the participants’ mean response to the nine items.

### 2.3. Data Analysis

Data were analyzed with SPSS version 27. Internal consistencies were examined, and variables were determined using item means. The distributions did not deviate from normal (skewness values ranged from −0.92 to 0.55, SE = 0.17). The study variables were described using means and standard deviations, and Pearson correlations were calculated between them. Independent t-tests were calculated for the study variables by gender. Pearson correlations and independent t-tests were calculated between the study variables and other demographic characteristics to identify background variables that needed to be controlled for. A multiple hierarchical regression was calculated to assess what trust, emotions, and perceptions of threat and risk contributed to preventive behavior. Multicollinearity was not found to be an issue, as the highest value of the variance inflation factor (VIF) was 1.77. All continuous variables were standardized, and their interactions with gender were defined and entered in a stepwise manner into the final step of the regression model. Significant interactions were interpreted with simple slopes [40]. Statistical power was calculated for a regression analysis with seven predictors and one interaction, using the G*Power software [41]. For this analysis, which included 187 participants and an explained variance percentage of R^2^ = 0.27 with α = 0.05, the achieved power level was 0.99. Finally, a multiple hierarchical regression was calculated to assess what trust, emotions, and threat and risk perceptions contributed to preventive behavior. Age and gender were entered in the first step as general control variables, trust was entered in the second step, emotions were entered in the third step, and threat and risk perceptions were entered in the fourth step. Furthermore, all continuous variables were standardized, and their interactions with gender defined and entered as the fifth step in the regression model.

## 3. Results

The participating physicians were asked about the extent to which they complied with the rules of hygiene and the preventive behavioral guidelines issued by the Ministry of Health in response to the COVID-19 outbreak. Most physicians responded that they complied fully (42%), very often (31%), or in general (20%) with the rules of hygiene at work. They tended to comply with these rules somewhat less stringently at home (full compliance—22%; very often—32%; in general—29%). They reported complying fully (27%), very often (34%), or in general (23%) with the behavioral guidelines issued by the Ministry of Health. Indeed, as shown in Table 2, the participants’ mean preventive behavior was rather high (M = 5.71range 1–7). The means for perceived threat and perceived risk ranged from moderate to high, the means for trust and positive emotions were moderate, and the mean for negative emotions was moderate–low. Positive correlations were found among compliance, threat and risk perceptions, and negative emotions. Negative and positive emotions exhibited a negative correlation with each other, while and positive emotions exhibited a negative association with perceived threat. Trust in the health system exhibited a negative association with negative emotions and a positive association with positive emotions. Trust exhibited a weak correlation with perceived threat. 

A series of *t*-tests was calculated to assess differences in the study variables by gender, as shown in Table 3. Significant differences were found, revealing that negative emotions were higher for female participants (3.37 versus 2.62, *p* < 0.001), while positive emotions were higher for male participants (3.94 versus 3.35, *p* < 0.001). In addition, the results shown in Table 3 indicate that threat and risk perceptions were significantly higher among female participants (5.31 versus 4.96, *p* < 0.05, for perceived threat; 4.75 versus 4.50, *p* < 0.05, for perceived risk). No gender differences were found for trust or for compliance with preventive behavior guidelines.

Pearson correlations and a series of t-tests were calculated between the study variables and other demographic characteristics (Table A1 in Appendix B). Age showed a negative association with negative emotions (r = −0.37, *p* < 0.001), a positive association with positive emotions (r = 0.24, *p* = 0.001), and a negative association both with perceived threat (r = −0.25, *p* < 0.001) and with perceived risk (r = −0.19, *p* = 0.010). Seniority in medicine was highly associated with age (r = 0.95, *p* < 0.001) and exhibited correlations with the study variables that were similar to those found for age.

Holding a managerial position was associated with emotions and perceived risk. Negative emotions were higher among physicians who did not hold managerial positions (M = 3.24, SD = 1.43) than among physicians in managerial positions (M = 2.30, SD = 0.95) (t (92.01) = 5.01, *p* < 0.001, t for unequal variances, Cohen’s d = 1.04), while positive emotions were higher among physicians in managerial positions (M = 4.12, SD = 1.07) than for those who did not hold such positions (M = 3.50, SD = 1.03) (t (198) = −3.41, *p* < 0.001, Cohen’s d = −0.48). Perceived risk was higher among physicians who did not hold managerial positions (M = 4.74, SD = 0.85) than among physicians in managerial positions (M = 4.30, SD = 0.77) (t (198) = 2.99, *p* = 0.003, Cohen’s d = 0.42) (see Table A2 in Appendix B). Finally, perceived risk was higher among non-Jewish participants (M = 5.18, SD = 0.75) than among Jewish participants (M = 4.53, SD = 0.83) (t (184) = 3.77, *p* < 0.001, Cohen’s d = 0.56) (see Table A3 in Appendix B).

The regression model described in Table 4 was found to be significant, with the study variables explaining 27 percent of the variance in compliance with preventive behavior. Trust, positive emotions, perceived threat, and perceived risk were found to be significant and positive, such that higher trust, more positive emotions, and higher perceived threat and perceived risk were associated with more compliant behavior. It is interesting to note that negative emotions were significant in the third step, but they lost their significance when perceived threat and perceived risk were added to the regression.

Table A4 (in Appendix B) shows the results of the multiple hierarchical regression for compliance, using the interaction between gender and perceived threat as a variable. The interaction between gender and perceived threat was found significant (β = −0.24, *SE* = 0.13, *p* = 0.015), adding 2% to the explained variance. Moreover, Table A4 shows a positive relationship between perceived threat and compliance for female participants (coefficient = 0.38, *t* = 3.42, *p* < 0.001) and a non-significant relationship for male participants (coefficient = 0.06, *t* = 0.65, *p* = 0.517). That is, a higher perceived threat was related to more compliant behavior among female physicians but not among male physicians.

## 4. Discussion

Healthcare workers, including physicians, are at the forefront of the battle against the COVID-19 pandemic [42]. HCWs have a high burden of work and are under constant stress [43]. Their high level of exposure to COVID-19 patients places them at greater risk of contracting the disease. The current study contributes to the existing literature by examining factors associated with physicians’ compliance with the MOH preventive behavior guidelines during the first wave of the pandemic in Israel, among them perceived risk, perceived threat, trust, and emotions.

### 4.1. Physicians’ Compliance MOH Behavioral Guidelines

The results show that most physicians complied fully or very often (73%) with the rules of hygiene at work, while they tended to comply with these rules less stringently at home (fully or very often 54%). In addition, most physicians reported complying fully or very often (61%) with MOH behavioral guidelines at work. These results suggest that although the level of preventive behaviors among physicians in Israel is generally satisfactory at work, their preventive measures at home are not optimal. Non-optimal preventive behavior among HCWs may be dangerous for them, their families, and their patients. These results are in line with the findings of a previous study conducted in Jordan [33]. Among Jordanian physicians, the mean work protection percentage score was 73.8%, while the mean home safety percentage score was lower (71.3%) [33]. The authors claimed that although the level of precautionary behaviors among medical doctors in Jordan was satisfactory in general, precautionary measures adopted at home were not optimum among doctors who live with high-risk groups or have morbidity risk factors [33].

### 4.2. Emotions

We are not aware of previous studies that examined the relationship between compliance behavior among HCWs and positive and negative emotions evoked during a pandemic. Our findings indicate that higher levels of positive emotions were associated with more compliant behavior among physicians. It is interesting to note that negative emotions were not significant in the final regression analysis in the presence of perceived threat and perceived risk. Therefore, our findings partially support H3 with respect to positive emotions. Our findings also confirm the predictions of the cognitive-social health information processing (C-SHIP) model [25], according to which affective states are related to preventive health behaviors.

### 4.3. Gender Differences

The current study contributes to the existing literature by focusing on gender differences in the context of emotions, perceived risk, perceived threat, and preventive behavior among physicians during the pandemic. Although we found no significant gender differences in the levels of preventive behavior, our findings point to significant gender differences in levels of emotions, perceived threat, and perceived risk. In particular, we found that negative emotions were higher among female physicians than among male physicians, while positive emotions were higher among male physicians than among female physicians. In addition, perceived threat and perceived risk were higher among women than among men. These results are compatible with H4 and in line with the findings of previous studies showing that, in the wake of major events, women generally report higher levels of fear and higher self-risk estimates than men [27,28,29,30]. Our findings are also in line with the results of Dryhurst et al. [32], who found that men have lower “COVID-19 risk perception” than women.

### 4.4. HBM Prediction—Perceived Threat

Our results confirm the HBM prediction that higher levels of perceived threat are related to higher levels of preventive behavior. This finding is compatible with H1 and in line with empirical findings in China showing that perceived severity and perceived susceptibility during the COVID-19 pandemic were important predictors of coronavirus prevention behaviors [17,43]. However, our analytical findings further reveal that higher perceived threat was related to more compliant behavior only among female physicians but not among male physicians. The results of the current study may imply that decision making among female physicians is based on emotions, while among male physicians, decision making may be more rational. While rationality-based decision making may appear to be more professional, emotion-based decision making may result in better performance, as demonstrated by Seo and Barrent [44].

### 4.5. Trust

Previous studies focused on public trust and other attributes of public attitudes and behavior. The current study adds to the literature by focusing on physicians’ trust in the MOH during the pandemic and their compliance with regulations. The findings reveal that higher levels of trust in the MOH and the healthcare system were associated with higher levels of compliance, which is compatible with H2. These results are in line with a previous study conducted among the public in 23 countries showing that higher trust in government regarding measures taken to control COVID-19 was significantly associated with higher levels of preventive behaviors [20]. Understanding the association between trust in government and the decision to comply with preventive behavior guidelines is important in controlling the spread of COVID-19.

### 4.6. Perceived Threat, Perceived Risk, Emotions, and Trust

Univariate analysis results (correlation analysis) show that higher perceived threat and perceived risk and higher levels of negative emotions were related to higher levels of preventive behavior and compliance with the guidelines. In addition, multivariate analyses (regression analyses) show that trust, positive emotions, and perceived risk were significant and positive, such that higher levels of these variables were associated with higher levels of preventive behavior. These results are in line with H1, H2, and H3 with respect to positive emotions.

### 4.7. Additional Measures to Prevent Infection—Vaccination Practice and Green Pass Policy

Data for the current study were obtained in April and May 2020. During this period, the only method to prevent infection was to adhere to the behavioral guidelines issued by the Ministry of Health. Toward the end of December 2020, about seven months after the data were collected for the current study, vaccinations against COVID-19 began being offered to medical teams in Israel, followed by populations at risk and then the rest of the population.

The vaccination campaign raised great hopes for battling the COVID-19 pandemic. In addition, following the vaccination campaign, a green pass policy was introduced in Israel during February 2021. This policy made entry into certain places conditional upon showing a vaccination certificate or a negative result on an up-to-date COVID-19 test. This policy has provoked discussions around the world regarding its necessity and the tension between individuals’ rights to choose for themselves whether to be vaccinated and the obligation to maintain public health [45,46].

The findings of the current study emphasize the importance of physicians’ trust in the healthcare system. A key component of this trust is the transparency of the information transmitted to physicians. In addition to providing medical care, physicians play an important role in encouraging their patients to be vaccinated. Therefore, healthcare workers must be provided comprehensive, reliable, and transparent information.

### 4.8. Limitations of the Study

This study has several limitations. First, the study is based on a self-reporting method, which may be subject to response bias and selectivity bias. Second, the non-randomized sampling technique limits the generalizability of our results. However, the current study does not seek to represent all Israeli physicians but rather to focus on a sample of physicians who work in healthcare institutions (hospitals and health maintenance organizations). The objective was to demonstrate the relationships among the research variables and to show their significance for the health of both physicians and patients. Third, it is worth mentioning that prior to and during the COVID-19 pandemic, Israel underwent several rounds of national elections, possibly affecting the population’s level of trust in government. In addition, the study was conducted only at one time point during the first wave of the COVID-19 pandemic, before the vaccines were available and before the green pass was introduced. Future studies should examine preventive behaviors adopted by HCWs over time and in different countries, including their attitudes toward vaccination and toward the green pass.

## 5. Conclusions

To conclude, HCWs’ level of trust in the health system and the level of their positive emotions at work during the pandemic are related to more careful decision making and higher compliance with guidelines. The link between emotions and decision making has been extensively researched in the past, but it has never been studied during a global epidemic among healthcare professionals. In light of this, we believe that the findings of the present study add to existing knowledge and can be used to understand the associations among the emotional state of physicians, their trust in the healthcare system, and their compliance with guidelines. These results may have international implications for policy makers in the Ministry of Health, as well as for managers of healthcare institutions in planning steps to enhance the levels of compliance with regulations.

While perceived threat and perceived risk may be unavoidable during an epidemic, the level of trust in the health system as well as the level of positive emotions that teams feel at work may promote more careful decision making and greater compliance to guidelines. Emotion-based decision making may also be associated with more burnout and higher levels of stress. Burnout may serve as an indicator that employees are no longer able to regulate their emotions adequately [47].

Burnout and negative emotions among HCWs may also have economic implications, including increased absences from work, and non-optimal decisions that can result in unnecessary expenses, including longer hospitalizations, complications, and unnecessary use of equipment. Therefore, healthcare institutions should take this into account by planning steps to enhance resilience and to provide a setting for venting and peer support.

Moreover, emphasis should be placed on emotional regulation and strengthening the sense of mission and pride among medical staff, along with maintaining and strengthening trust in the health system by providing reliable and transparent information. All these steps to maintain the physical and mental health of the healthcare staff are important for improving their function and maintaining public health.

## Figures and Tables

**Table 1:** Participants’ demographic characteristics by gender.

	Range	Total Sample (n = 187)	Male (n = 92)	Female (n = 95)	
Gender (%)		100.00	49.20	50.80	-
Age (years) M (SD)	27–77	41.27 (10.60)	42.91 (11.31)	39.67 (9.67)	t = 2.11, *p* = 0.04, d * = 0.31
Seniority in medicine (years) M (SD)	0.5–50	12.54 (11.59)	13.95 (12.37)	11.16 (10.66)	t = 1.64, *p* = 0.10, d = 0.24
Status (%)	Physician	97.00	98.90	94.70	-
	Medicine resident	3.00	1.10	5.30	
Managerial position (%)	Yes	20.50	22.80	17.90	Z = 0.84, *p* = 0.40, d = 0.12
Religion (%)	Jewish	86.00	80.40	91.5	Z = 2.17, *p* = 0.03, d = 0.31
Country of birth (%)	Israel	75.30	76.10	74.50	Z = 0.26, *p* = 0.79, d = 0.04
Marital status (%)	Married/ in an intimate relationship	88.80	89.10	88.40	Z = 0.15, *p* = 0.87, d = 0.02
	Single, divorced	11.20	10.90	11.60	
Economic status (%)	Average and below	17.50	15.60	19.40	Z = 0.68, *p* = 0.49, d = 0.10
	Above average	82.50	84.40	80.60	
Religiosity (%)	Secular	71.00	64.10	77.70	χ^2^ = 4.47
	Partly religious	16.60	21.70	11.70	*p* = 0.11
	Religious	12.40	14.10	10.60	d = 0.30
Current main work place (%)	Hospital	90.90	84.80	77.70	Z = 1.24, *p* = 0.21, d = 0.17
	Other (e.g., community service)	9.10	15.20	22.30	

Note. No differences were calculated for gender, as gender describes the sample. No differences were calculated for status as the variable had low variance. d * = Cohen’s d effect size.

**Table 2:** Means, standard deviations, and intercorrelations for the study’s variables (*N* = 200).

	Cronbach’s Alpha	M	SD	2.	3.	4.	5.	6.
Trust	0.71	3.74	0.96	−0.34 ***	0.24 ***	−0.14 *	−0.07	0.11
Negative emotions	0.91	3.04	1.40		−0.28 ***	0.52 ***	0.45 ***	0.18 **
Positive emotions	0.76	3.62	1.07			−0.23 ***	−0.12	0.09
Perceived threat	0.75	5.15	1.00				0.58 ***	0.36 ***
Perceived risk	0.87	4.65	0.85					0.43 ***
Compliance-Preventive Behavior	0.72	5.71	1.00					

* *p* < 0.05, ** *p* < 0.01, *** *p* < 0.001. Range 1–7.

**Table 3:** Means, standard deviations, and t values for the study variables by gender.

	Male		Female		t = (185)	Cohen’s d	*p*
	M	SD	M	SD			
Trust	3.79	0.94	3.76	0.96	0.22	0.03	0.82
Negative emotions	2.62	1.30	3.37	1.34	−3.89	−0.57	<0.001
Positive emotions	3.94	1.10	3.35	0.99	3.85	0.57	<0.001
Perceived threat	4.96	1.08	5.31	0.91	−2.33	−0.35	0.02
Perceived risk	4.50	0.95	4.75	0.74	−2.03	−0.31	0.04
Compliance-Preventive Behavior	5.65	0.92	5.71	1.09	−0.41	−0.06	0.68

Note. t for unequal variances: perceived threat df = 177.32; perceived risk df = 171.85.

**Table 4:** Multiple hierarchical regression for compliance with trust, emotions, perceived threat, and perceived risk as variables (*N* = 187).

	Model 1 β (*SE*)	Model 2 β (*SE*)	Model 3 β (*SE*)	Model 4 β (*SE*)
Age	0.05 (0.01)	0.03 (0.01)	0.11 (0.01)	0.14 (0.01)
Gender-male	−0.02 (0.15)	−0.02 (0.15)	0.01 (0.15)	0.02 (0.14)
Trust		0.15 * (0.08)	0.21 ** (0.08)	0.18 ** (0.07)
Negative emotions			0.33 *** (0.06)	0.08 (0.06)
Positive emotions			0.17 * (0.07)	0.18 ** (0.07)
Perceived threat				0.20 * (0.08)
Perceived risk				0.36 *** (0.09)
Adj. R^2^	0.001	0.01 *	0.09 ***	0.27 ***

F(7, 179) = 10.86, *p* < 0.001. * *p* < 0.05, ** *p* < 0.01, *** *p* < 0.001.

## Data Availability

Data from authors will be available upon request.

## Appendix A. The Questionnaire

**Part a:** Trust in the healthcare system

Participants were asked to indicate their level of agreement with the following items on a 7-point scale (1 = do not agree at all, 7 = fully agree):

1. The Israeli health system routinely performs its work well.
2. The Ministry of Health is managing the coronavirus crisis in an excellent and professional manner.
3. The guidelines issued by the Ministry of Health to the public regarding the COVID-19 pandemic are exaggerated (reversed score).
4. The way the COVID-19 crisis is being managed is motivated by political motives (reversed score).
5. The Ministry of Health does not take sufficient measures to protect the medical staff (reversed score).
6. I have confidence in the medical information published by the Ministry of Health.
7. The Ministry of Health will vaccinate medical staff only if the vaccine is found to be effective and safe.
8. I have confidence in the medical information published by the Ministry of Health regarding the safety of the COVID-19 vaccine.
9. The organization where I work is being run well during the COVID-19 epidemic.

**Part b**: Compliance with MOH regulations

Participants were asked to indicate how frequently they perform the following actions during the coronavirus crisis on a Likert-scale ranging from 1 = not at all to 7 = always.

1. More meticulous than usual in complying with MOH-issued COVID-19 regulations.
2. More meticulous than usual in adhering to hygiene guidelines at work.
3. More meticulous than usual in adhering to hygiene guidelines at home.

**Part c**: Perceived Threat

Participants were asked to indicate the levels of their agreement with the following 7 items, on a 7-point scale ranging from 1 = “strongly disagree” to 7 = “strongly agree”:

1. The thought of getting COVID-19 scares me.
2. I worry a lot about getting COVID-19.
3. My chances of getting COVID-19 are high.
4. COVID-19 can be a serious disease and can cause medical complications and even death.
5. Working with numerous people each day increases my chances of getting COVID-19 and infecting my family.
6. If I get COVID-19, my family will be nervous and scared.
7. If I get COVID-19, my functioning will be impaired.

**Part d**: Perceived risk during the COVID-19 pandemic

Participants were asked to indicate the level of danger they feel regarding the following items, on a 7-point scale ranging from 1 = “not dangerous at all” to 7 = “extremely dangerous”:

1. working at a hospital.
2. working in the community (community clinics).
3. treating a patient suspected of having COVID-19 without protective equipment.
4. treating a COVID-19 patient without protective equipment.
5. touching public surfaces without gloves.
6. not washing hands after touching public surfaces.
7. meeting family members; shopping for food and drugs; using public transportation.

**Part e**: Positive and negative emotion levels

Participants were asked to indicate the levels of negative and positive emotions they felt during the last week, on a 7-point scale ranging from “not at all” (1) to “extremely” (7).

The negative emotions included fear, anger, anxiety, stress, nervousness, bad mood, blame, and frustration.

The positive emotions included enthusiasm, relaxation, strength, “sense of mission”, pride, and activism.

**Part f**: Socio-demographic information and personal information

Gender; age; marital status; nationality; degree of religious observance (secular/somewhat religious/religious); country of birth (Israel/other); household income (1 = above average, 5 = much lower than average); position at work (managerial/non-managerial); place of work (hospital/community service); experience as a physician (years).

## Appendix B

**Table A1:** Pearson correlations between the study variables and age and seniority in medicine (*N* = 187).

	Age	Seniority in Medicine
Trust	0.10	0.10
Negative emotions	−0.37 ***	−0.36 ***
Positive emotions	0.24 **	0.21 **
Perceived threat	−0.25 ***	−0.23 **
Perceived risk	−0.19 **	−0.16 *
Preventive Behavior	0.05	0.06

* *p* < 0.05, ** *p* < 0.01, *** *p* < 0.001.

**Table A2:** *t*-tests for the study variables by managerial position (*N* = 200).

	Managerial Position	No Managerial Position	t (198)	*p*
Trust	3.82 (1.02)	3.72 (0.95)	−0.58	0.560
Negative emotions	2.30 (0.95)	3.24 (1.43)	5.01	<0.001
Positive emotions	4.12 (1.07)	3.50 (1.03)	−3.41	<0.001
Perceived threat	4.90 (0.89)	5.22 (1.02)	1.80	0.073
Perceived risk	4.30 (0.77)	4.74 (0.85)	2.99	0.003
Preventive Behavior	5.85 (0.98)	5.67 (1.00)	−1.06	0.292

For negative emotions: df = 92.01.

**Table A3:** *t*-tests for the study variables by ethnicity (*N* = 186).

	Arab	Jewish	t (184)	*p*
Trust	4.00 (0.75)	3.75 (0.97)	1.26	0.210
Negative emotions	2.97 (1.30)	2.98 (1.35)	−0.04	0.965
Positive emotions	3.54 (1.20)	3.67 (1.05)	−0.56	0.576
Perceived threat	5.24 (0.97)	5.12 (1.02)	0.60	0.551
Perceived risk	5.18 (0.75)	4.53 (0.83)	3.77	<0.001
Preventive Behavior	5.77 (1.33)	5.66 (0.95)	0.51	0.612

**Table A4:** Multiple hierarchical regression for preventive behavior, with the interaction of gender and perceived threat (*N* = 187).

	Total		Female			Male		
	β (SE)	*p*	Coefficient	t	*p*	Coefficient	t	*p*
(Gender) x (perceived threat)	−0.24 (0.13)	0.015	0.38	3.42	<0.001	0.06	0.65	0.517
